# Are visual working memory and episodic memory distinct processes? Insight from stroke patients by lesion-symptom mapping

**DOI:** 10.1007/s00429-021-02281-0

**Published:** 2021-04-29

**Authors:** Selma Lugtmeijer, Linda Geerligs, Frank Erik de Leeuw, Edward H. F. de Haan, Roy P. C. Kessels, on behalf of The Visual Brain Group, Anouk R. Smits, Ben A. Schmand, Edward H. F. de Haan, Frank Erik de Leeuw, Gert jan Luijckx, H. Steven Scholte, Joke M. Spikman, L. Jaap Kappelle, Linda Geerligs, Martine J. E. van Zandvoort, Matthan W. A. Caan, Matthijs A. H. L. L. Raemaekers, Mathias Prokop, Nick F. Ramsey, Nikki A. Lammers, Nils S. van den Berg, Noor Seijdel, Paul J. Nederkoorn, Rients B. Huitema, Bob Kentridge, Roy P. C. Kessels, Selma Lugtmeijer, Yair Pinto

**Affiliations:** 1 Donders Institute for Brain, Cognition and Behaviour, Radboud University, Nijmegen, The Netherlands; 2 University of Amsterdam, Amsterdam, The Netherlands; 3 Department of Neurology, Radboud University Medical Center, Nijmegen, The Netherlands; 4 Department of Medical Psychology, Radboud University Medical Center, Nijmegen, The Netherlands; 5 Vincent Van Gogh Institute for Psychiatry, Venray, The Netherlands; 6 Donders Institute for Brain, Cognition and Behaviour, Centre for Cognition, Neuropsychology and Rehabilitation Psychology, Radboud University, PO Box 9101, 6500 HB Nijmegen, The Netherlands

**Keywords:** Activated long-term memory, Episodic memory, Lesion-symptom mapping, Multicomponent model, Stroke, Working memory

## Abstract

Working memory and episodic memory are two different processes, although the nature of their interrelationship is debated. As these processes are predominantly studied in isolation, it is unclear whether they crucially rely on different neural substrates. To obtain more insight in this, 81 adults with sub-acute ischemic stroke and 29 elderly controls were assessed on a visual working memory task, followed by a surprise subsequent memory test for the same stimuli. Multivariate, atlas- and track-based lesion-symptom mapping (LSM) analyses were performed to identify anatomical correlates of visual memory. Behavioral results gave moderate evidence for independence between discriminability in working memory and subsequent memory, and strong evidence for a correlation in response bias on the two tasks in stroke patients. LSM analyses suggested there might be independent regions associated with working memory and episodic memory. Lesions in the right arcuate fasciculus were more strongly associated with discriminability in working memory than in subsequent memory, while lesions in the frontal operculum in the right hemisphere were more strongly associated with criterion setting in subsequent memory. These findings support the view that some processes involved in working memory and episodic memory rely on separate mechanisms, while acknowledging that there might also be shared processes.

**Supplementary Information:**

The online version contains supplementary material available at 10.1007/s00429-021-02281-0.

## Introduction

Working memory and episodic memory are two different processes, although the nature of their interrelationship is debated. The multicomponent perspective on human memory function (e.g., Squire 2004) is based on clinical cases with specific memory deficits and has been supported by neuroimaging studies that indicated a frontoparietal network to be involved in working memory processes (D’Esposito et al. 2000; Rottschy et al. 2012), whereas the medial temporal lobe is associated with long-term memory processes (Spaniol et al. 2009; Squire 1992). In contrast, other memory models that distinguish between different processes for short-term and long-term memory do not necessarily imply different neural mechanisms but describe working memory as activated portion of long-term memory (e.g.,Atkinson and Shiffrin 1971; Cowan 1988). According to this view, memory representations can be in a temporarily activated state so that they are easily accessible. This activated state is limited to items in the focus of attention.

There is accumulating evidence showing that brain regions typically associated with long-term memory, such as the hippocampus, are active during working memory and that frontal and parietal regions are active during long-term memory (reviewed in Ranganath and Blumenfeld 2005). However, only a few studies take into account that activation during a working memory task might actually reflect long-term memory formation rather than working memory processing. The studies that do, report mixed results concerning parahippocampal and hippocampal involvement in working memory processes (Axmacher et al. 2008; Bergmann et al. 2015, 2016; Zanto et al. 2015).

The key distinction between the multicomponent view of memory and the activated long-term memory view is the need for a separate copy of information, or a set of temporary pointers to relevant long-term memory, in a distinct working memory store (D’Esposito and Postle 2015; Baddeley et al. 2019; Cowan 2019; Norris 2017, 2019; Oberauer 2009; Shallice and Papagno 2019). As working memory and episodic memory are predominantly studied in isolation, it is unclear whether they crucially rely on different neural substrates. Patients with brain lesions might give insight as the two theoretical models make different predictions for patients with brain injury. According to the multicomponent model of memory, working memory and episodic memory performance can be separately affected by brain lesions and have a distinct neural profile as two separate representations are formed. Based on the theory of activated long-term memory, direct and delayed memory rely on the same representations. Therefore, neural correlates of working memory and episodic memory are expected to partly overlap. Two behavioral profiles fit this theory of activated long-term memory. One, impaired performance on both the working memory and episodic memory task due to a failure in rapid new learning. Two, impaired performance on only the episodic memory task that can be explained by a failure to consolidate information due to time-based decay or interference.

To date, no study directly compared working memory and long-term memory processing in patients with brain lesions. We thus employed an *N*-back task with easy to name stimuli to assess working memory (Lugtmeijer et al. 2019). In this way, we avoided the processing of complex stimuli which might engage long-term memory processing even when the retention interval is short (Jeneson and Squire 2012), without inducing a ceiling effect (Axmacher et al. 2008) that might arise from a match-to-sample design with simple stimuli. The *N*-back task was followed by an unexpected subsequent memory task in which participants had to indicate whether an object is on the same location of the screen as during the *N*-back task. The encoding phase is the same for both tasks as encoding takes place during the first presentation of the object during the working memory task. During this first presentation, an object is bound to both serial order and spatial location. Working memory performance is based on maintenance of this bound information for object and order, while performance on the subsequent memory task is based on recollection of spatial information bound to an object.

Our first goal was to determine how working memory and episodic memory performance are related in an unselected cohort of stroke patients. Our second goal was to investigate unique and shared lesion locations associated with working memory and episodic memory. We used multivariate lesion symptom mapping and atlas-based lesion symptom mapping to identify on voxel- and ROI-level areas that contribute to memory performance.

## Materials and methods

### Study design

This study is part of the functional architecture of the brain for vision (FAB4V) study, a multi-center prospective cohort study on vision and cognition after ischemic stroke in adults aged 18 through 90 years. Patients were admitted between September 2015 and December 2019 to one of the following hospitals in The Netherlands: Amsterdam University Medical Center (Amsterdam UMC), Radboud University Medical Center (Radboudumc) in Nijmegen, University Medical Center Groningen (UMCG), University Medical Center Utrecht (UMCU), Onze Lieve Vrouwe Gasthuis (OLVG), Maasziekenhuis Pantein, Rijnstate, Ommelander Ziekenhuis Groep, St. Antonius Ziekenhuis, and Diakonessenhuis. Assessment took place at one of the four academic hospitals. The Medical Review Ethics Committee Utrecht approved the study (30-06-2015), and written informed consent was obtained from all participants prior to participation.

Patients were identified based on their medical records at admission to the hospital and in consultation with their treating neurologist or nurse practitioner approached for participation. Ischemic stroke was defined as focal neurological deficit persisting > 24 h. Inclusion criteria: diagnosis of ischemic stroke made by an expert neurologist, age between 18 and 90, sufficient Dutch language skills to understand task instructions. Exclusion criteria: hemorrhagic stroke, cerebral venous sinus thrombosis, pre-existing cognitive decline (Score ≥ 3.6 on the Informant Questionnaire on Cognitive Decline in the Elderly [IQCODE; Jorm, 2004], assessing everyday cognitive functioning before the stroke compared with 10 years earlier, as reported by an informant, e.g., child or spouse) or dementia, pre-existing visual impairment, psychiatric disorder and severe aphasia. Examination took place between 2 weeks and 6 months post stroke.

Patients who participated between July 2016 and March 2019 at the Radboudumc, Amsterdam UMC, and UMCG were recruited for the memory subgroup. These patients were tested more extensively on memory than the standard neuropsychological assessment of the cohort study.

A stroke-free control group existed of 29 older adults, aged 62–90 (*M* = 72.1, SD = 6.8, 13 men). There was no difference in level of education (*t* (107) = 1.19, *p* = 0.24), but the controls were significantly older than the patients (*F *(90.77) = 5.860, *p* < 0.001). We deliberately opted for a control group of older adults to obtain more variance in behavioral performance and to minimize ceiling performance, which may occur in younger healthy controls. Controls did not have a history of neurological disease or cognitive decline (self-report). Controls were recruited from social networks and received monetary compensation for their participation.

### Memory assessment

**Fig. 1:**
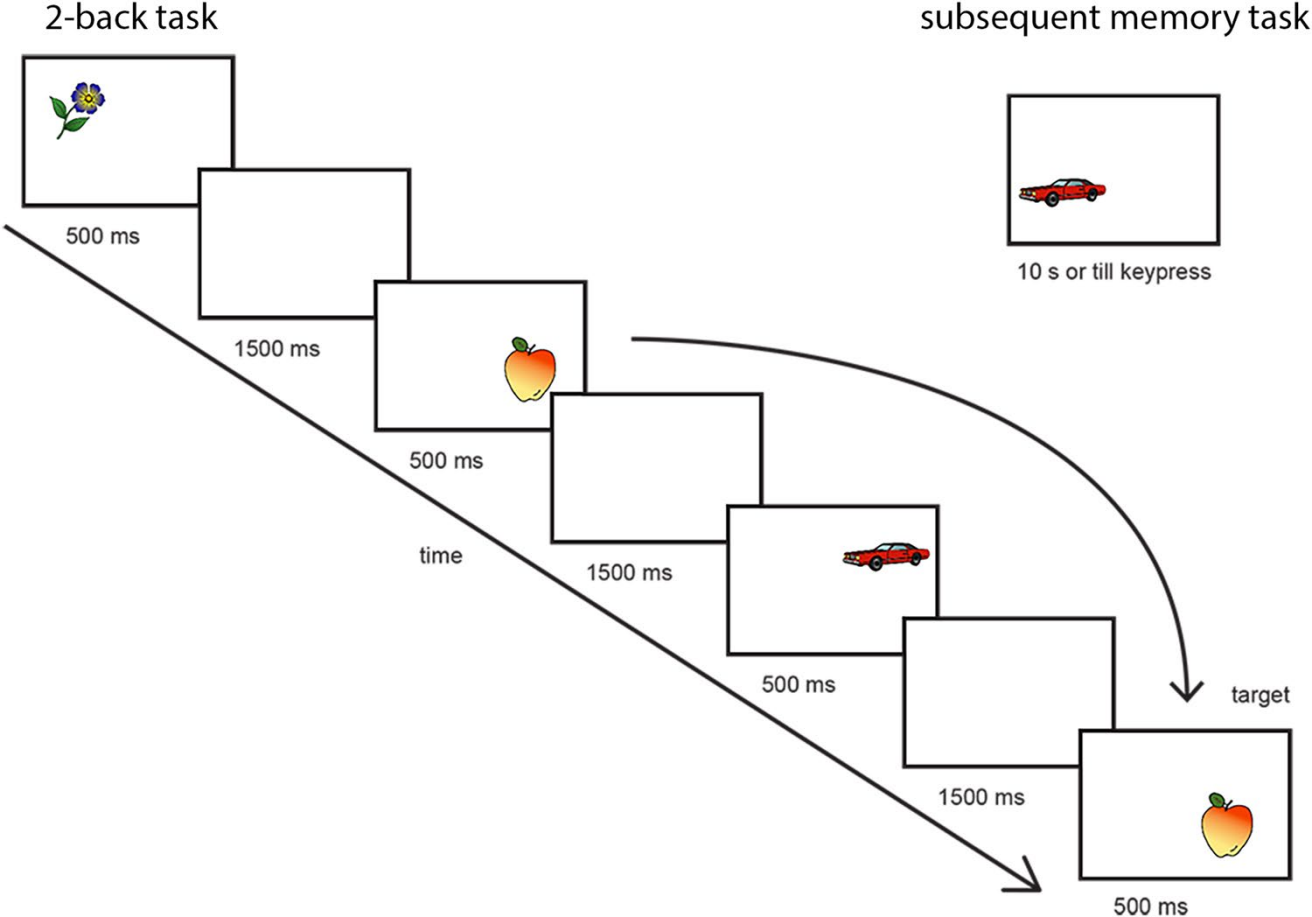
Task design. 2-back task from left to right. In the upper right corner, a stimulus example from the subsequent memory task in which the correct answer is “false” as the car in is the lower left corner while it was in the upper right corner during the 2-back task

To assess visual working memory, an *N*-back task with common objects was employed (for more details on the task see Lugtmeijer et al. 2019). In short, during the 2-back task, stimuli are presented in serial presentations and participants identify stimuli that are identical to a stimulus presented two trials before. This requires temporal-order binding. Stimuli were 50 easy-to-name objects that were presented in each of the four corners of the screen. Presentation time was 500 ms followed by an interstimulus interval of 1500 ms. A schematic overview of the task is represented in Fig. 1. The task consisted of 5 blocks of 20 trials with four targets (20%) per block. Every object was presented twice within the same block and the second presentation was always in the same location as the first. Participants responded only to targets by pulling a joystick towards them. In case of physical limitations, participants could respond verbally.

Directly following the 2-back task participants completed a surprise subsequent recognition memory test for assessing episodic memory function. Here, participants had to indicate whether an object was presented in the same corner of the screen now, as during the 2-back task. All objects from the 2-back were presented once, no new items were added. Out of 50 objects, 20 were presented at the same location as before (targets). Half of these targets had also been targeted in the working memory task. This task relies on visuospatial binding. The stimuli were presented until the participant responded, within a limit of 10 s (see Fig. 1).

For both tasks four types of responses were possible: hits, misses, false alarms, and correct rejections. How well participants could discriminate targets from nontargets was expressed as d-prime (*d′*), higher scores indicate better performance. Response criterion (*c*) reflects an overall preference to answer yes (target) or no (non-target). Positive values indicate a conservative response bias, while negative values reflect a liberal response bias.

### Imaging data acquisition

Participants underwent a 3-T magnetic resonance imaging scan, at the Radboudumc and UMCG on the Siemens Magnetom Prisma, at the Amsterdam UMC and UMCU on the Philips R5. For the Siemens scanner, sequence details were as follows: T2 FLAIR (TI = 1650 ms, TR = 4800 ms, TE = 484 ms, [FOV] = 280 mm, voxel size 0.9 × 0.9 × 0.9mm^3^). For the Philips scanner, sequence details were: T2 FLAIR (TI = 1650 ms, TR = 4800 ms, TE = 253 ms, [FOV] = 250 mm, voxel size 1.12 × 1.12 × 0.56mm^3^).

### Lesion segmentation and preprocessing

Lesions were delineated semi-automatically or fully manually in native space using ITK-snap software (Yushkevich et al. 2006) on the axial slices of the FLAIR, checked in sagittal and coronal directions. Hyper-intensities surrounding the lesion indicating additional white matter degeneration and gliosis were included as a part the lesion. Lesions were delineated by three researchers. To check for interrater reliability eight scans (10%) were randomly selected and lesions were delineated by the researchers independently. A score was calculated as the number of voxels selected by all three raters, in reference to the mean of number voxels selected per rater (Neumann et al., 2009). The mean overlap for all three raters was 81.3% (range 69.8—91.1%). In case of doubt for specific scans, a neurologist or radiologist was consulted.

The FLAIR and binary lesion mask were normalized to an older adult MNI template using the unified segmentation/normalization algorithm implemented in SPM12 (Crinion et al. 2007; Rorden et al. 2012). For unilateral lesions, enantiomorphic normalization was applied as this method has been shown to be vastly superior to cost function masking (Nachev et al. 2008). For bi-lateral lesions, cost function masking was applied. Normalisation was inspected for all individuals by visually comparing the normalized lesion mask overlaid on the FLAIR in MNI space to the lesion mask and FLAIR in native space. Segmentations in MNI space were manually corrected when necessary.

### Multivariate lesion symptom mapping

Multivariate LSM analyses were performed using support vector regression (SVR-LSM) (Zhang et al. 2014) with a toolbox that allows for the adding of covariates and different lesion volume correction methods (DeMarco and Turkeltaub 2018). Multivariate LSM has a higher sensitivity and specificity for detecting the lesion-behavior relations by considering intervoxel correlations compared to univariate lesion-behavior mapping methods (Zhang et al. 2014). Settings of hyperparameter values, with a cost of 30 and gamma of 5, were kept in line with recommendations from the original publication (Zhang et al. 2014). Analyses were conducted with and without lesion volume correction. Lesion volume was corrected for by regressing lesion volume on the behavioral scores and lesion data in each voxel. This was based on recommendations by DeMarco and Turkeltaub (2018), who showed that regressing lesion volume on both behavioral and lesion data addresses the bias of lesion volume most effectively, without being overly conservative, while at the same time being more conservative than the commonly used method of direct total lesion volume control (dTLVC). Only voxels that were lesioned in a preset number of participants are included in the analyses, a correction known as ‘sufficient lesion affection’. In accordance with previous studies, we set the threshold at 5% of the whole sample (Sperber and Karnath 2017), which translates to voxels lesioned in at least four participants. Permutation testing (10,000 permutations) was used for testing statistical significance for the β values, with a voxelwise threshold of *p* < 0.005, and a clusterwise threshold of *p* < 0.05, only including clusters larger than 50 voxels. Age, education level (scored in categories based on the Dutch educational system, range 1–7, low to highly educated; Verhage 1964), interval between stroke and assessment, and scanner were regressed out from the behavioral scores and lesion data.

### Atlas-based lesion-symptom mapping

For atlas-based LSM, the statistical lesion analysis software NiiStat was used (https://github.com/neurolabusc/NiiStat). The atlas-based analysis we used relies on the cumulative lesion burden in a specific region, instead of investigating lesions on a voxel-wise basis. This has the advantage of effectively increasing the number of areas that have sufficient coverage across participants, assuming that lesions in nearby voxels affect behavior in the same way. In addition, univariate voxel-wise LSM is conservative due to strict multiple testing corrections, in a ROI-based approach this effect is reduced. For white-matter regions of interest (ROIs), we used the CAT atlas containing 32 ROIs (Catani and De Schotten 2008; https://www.natbrainlab.co.uk/). For gray-matter, ROIs the corrected Glasser atlas that defines 360 ROIs was used (Glasser et al. 2016; https://identifiers.org/neurovault.collection:1549). Analyses were conducted with and without lesion volume correction. Lesion volume control in NiiStat is based on regressing the lesion volume with the behavioral variable. We adapted the code to be able to regress lesion volume on both the behavioral scores and ROI-based lesion data, in line with our multivariate LSM analysis. Only ROIs included in the lesion masks of at least four participants were analysed. Permutation testing to correct for multiple testing, was set to 10,000 permutations at *p* > 0.05. Age, education level, interval between stroke and assessment, and scanner were included as covariates and the toolbox was adjusted to regress these on the behavioral and lesion data. To test if effects were specific for the working memory or subsequent memory task, the performance on the other measure was included in a subsequent analysis as covariate.

### Track-based lesion-symptom mapping

A track-based LSM analysis was conducted to validate the results from the multivariate and atlas-based LSM analyses where it concerned white matter tracts. Lesions from each patient were mapped onto tractography reconstructions of white matter pathways obtained from a group of 47 healthy controls (Rojkova et al. 2016). The Tractotron software (http://www.brainconnectivitybehaviour.eu) provides a probability of a lesioned voxel intersecting a specific tract. If the probability of a lesioned voxel overlapping with a tract was higher than 50%, the tract was considered disconnected, otherwise it was considered preserved (Thiebaut de Schotten et al. 2014). A Mann–Whitney *U* test was used to compare patients with disconnection and without on the behavioral measures. A second measure was the correlation with the severity of disconnection expressed as the proportion of lesioned voxels overlapping with the tract divided by the total number of voxels in that tract (Thiebaut de Schotten et al. 2014). The significance of these correlations was assessed by permutation testing with 10,000 permutations, in line with the LSM analyses. Only associations between tracts identified by the multivariate and atlas-based LSM analyses were included. The effects of age, education, interval between stroke and assessment, and scanner were regressed out of the MRI and behavioral data.

### Statistical analyses

To test how representative our memory subgroup was for the total cohort, we tested for group differences in baseline characteristics between patients in the memory subgroup and those in the total cohort with an independent sample *t* test, a Mann–Whitney *U* test, or Pearson *χ*^2^ test, when appropriate. Two-tailed *p* values < 0.05 were considered statistically significant. Performance on the experimental tasks was compared to an aging, stroke-free, control group with independent sample *t* tests.

Associations between working memory and episodic memory performance, and performance and lesion volume, were tested with partial correlations (Pearson’s *r*), adjusting for age and education level. Bayesian pairwise correlations were used to test the strength of the support for the null-hypothesis or alternative hypothesis. An ANOVA with age and education as covariates, was used to test the difference between patients and controls on discriminability and criterion in working memory and episodic memory. The association between lesion volume and behavioral measures was assessed with correlations. Before computing the correlations, variance due to age, education, interval between stroke and assessment, and scanner was regressed out of the behavioral measures and lesion volume. Because lesion volume was not normally distributed, the significance of these correlations was assessed by permutation testing with 10,000 permutations, in line with the LSM analyses. For criterion, both positive and negative values indicate a larger response bias and therefore less optimal response patterns. The value 0 indicates no bias, positive values indicate a conservative bias and negative values a liberal bias. Therefore, we first tested whether lesion volume was associated with a larger response bias, independent of direction, using the absolute values of criterion. Only if that was the case, we tested the direction of the effect using the continuous measure of criterion. This same two-step procedure was applied in the LSM analyses. To test for specific deficits, we selected patients with scores 2 SD below the control group mean for each memory task and investigated how many of patients performed low on both tasks.

## Results

### Participants

Of the 289 patients included in the cohort, a subset of 105 was recruited for participation in the memory study, the memory subgroup. Twenty-four patients were excluded from all analyses due to no MRI (*N* = 4), no FLAIR sequence (*N* = 8), or no lesion visible (*N* = 12). This resulted in a final sample of 81 patients. Patients in the memory subgroup did not differ from other patients in the cohort on descriptive variables, stroke characteristics, vascular risk factors, or memory function as measured by standard assessment (Table 1). The only difference between the patients included in the subgroup and those who were not is the number of patients with no MRI or no lesion visible on MRI, as that was an exclusion criterion. None of the patients in the memory group had neglect based on the Bells Test (Gauthier et al. 1989). Three patients demonstrated a visual field deficit, one in the right visual field, the other two in the lower-left quadrant. All patients reported to be able to perceive the stimuli of the memory task in all corners of the screen and none of the patients with a visual field deficit performed deviant on the memory tasks.

**Table 1:** Descriptives of patients in the memory subgroup and other patients in the cohort

	Memory subgroup	Other patients	*P*
No.	81	208	NA
Men no. (%), *χ*^2^	61 (75)	138 (67)	0.15
Age *M* (*SD*) [range], *t* test	59.8 (12.5) [20–89]	61.0 (13.4) [19–89]	0.46
Handedness r:l:a:u, (*r* %), *χ*^2^	70:9:1:1 (86)	171:20:5:12 (82)	0.78
Education *Median* [range], *χ*^2^	5 [2–7]	5 [1–7]	0.84
IQ estimate^a^ M (SD), *t* test	100.5 (15.8)	103.5 (13.1)	0.12
HADS depression M (SD), *t* test	3.31 (2.88)	3.82 (3.86)	0.26
HADS anxiety *M* (*SD*), *t* test	3.83 (3.17)	4.82 (4.12)	0.05
Neglect^b^ *N*	0	7	NA
Visual field deficit^c^ *N*	3	29	NA
Previous stroke n:y:u, *χ*^2^	62:13:6	150:46:12	0.27
Hemisphere l:r:b:c:a, *χ*^2^	35:32:12:2:0	58:60:28:8:43	< 0.001
Hypertension no. (%), *χ*^2^	33 (41)	80 (38.5)	0.94
Diabetes I/II no. (%), *χ*^2^	1 (1.2)/10 (12.3)	2 (1.0)/24 (11.5)	0.96
Hypercholesterolemia no. (%), *χ*^2^	26 (32.1)	44 (21.2)	0.09
Interval in days^d^ *M* (*SD*), *t* test	53.3 (26.2)	61.4 (35.7)	0.07

*NA* not applicable, *r* right, *l* left, *a* ambidextrous, *u* unknown, *y* yes, *n* no, *b* bilateral, *c* cerebellar/brain stem, *a* no MRI or no lesion on MRI

^a^Premorbid IQ estimated with the Dutch version of the National Adult Reading Test

^b^Neglect based on the performance on the Bells Test

^c^Computer-based visual field test: participants were instructed to indicate when they perceived a stimulus-dot presented in a quadrant of the screen, or in the middle

^d^Interval between stroke and assessment

### Behavioral performance

**Fig. 2:**
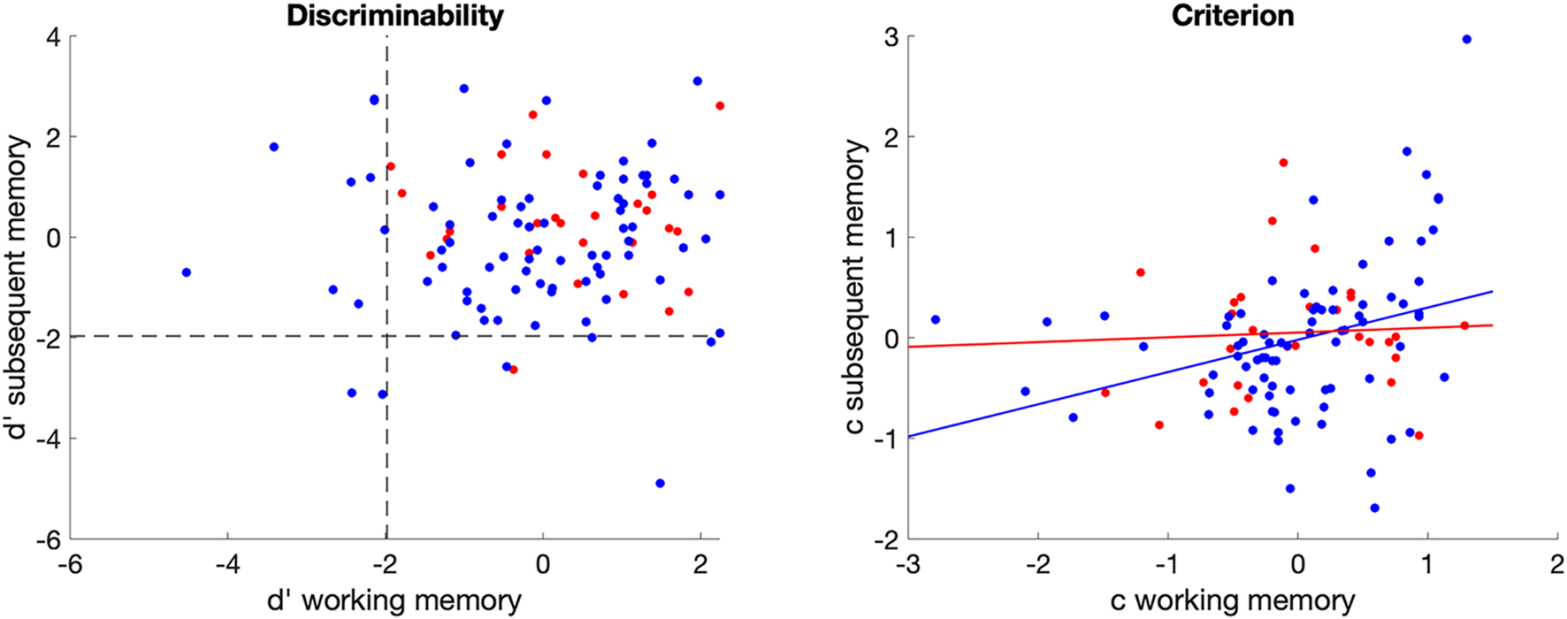
Performance from patients and controls with the 2-back task on the x-axis and subsequent memory task on the y-axis, **a** discriminability (*d’*) with reference lines at 2 *SD* below average performance based on the control group, **b** criterion (*c*)

Partial correlations were used to determine the relationship between performance on the working memory and episodic memory task, whilst controlling for age and education and in patients for interval between stroke and assessment. In patients, there was no significant partial correlation for discriminability, *r* (79) = −0.03, *p* = 0.82. There was a significant correlation for criterion, *r* (79) = 0.30, *p* = 0.01. Bayesian pairwise correlations corrected for age, education, and interval between stroke and assessment, based on a hypothesis of positive correlation, gave moderate evidence in favor of the null hypothesis for no correlation in discriminability, BF_10_ = 0.12, and strong support for a correlation between criterion on both tasks, BF_10_ = 8.46 (Jarosz and Wiley 2014). In the control group, both discriminability and criterion did not correlate (*d’*: *r* (29) = −0.10, *p* = 0.61, BF_10_ = 0.16; *c*: *r *(29) = 0.13, *p* = 0.49, BF_10_ = 0.43, Fig. 2).

A one-way ANOVA corrected for age and education, shows that at group-level patients had lower discriminability than controls for the 2-back task (*F* (1, 106) = 5.80, *p* = 0.02), but not for the subsequent memory task (*F* (1, 104) = 1.63, *p* = 0.21). For absolute response bias, mean scores for both groups were similar in the 2-back task (*F* (1, 106) = 0.31, *p* = 0.58), in the subsequent memory task patients showed a stronger bias (*F*(1, 104) = 4.61, *p* = 0.03). This stronger response bias in the subsequent memory task is in both directions (more liberal and more conservative) as there is no difference between patients and controls for the continuous measure of criterion (*F* (1, 103) = 0, *p* > 0.99). Interval between stroke and assessment only correlated with the absolute score on criterion setting on the subsequent memory task (*r* (79) = 0.26, *p* = 0.02).

Further investigation to get insight in system-specific deficits in patients who performed worse than average (two SD below the mean of the control group) in terms discriminability, showed that nine patients only had an impairment on the 2-back task, four only on the subsequent memory task, and two on both tasks (Fig. 2).

### Lesion distribution

**Fig. 3:**
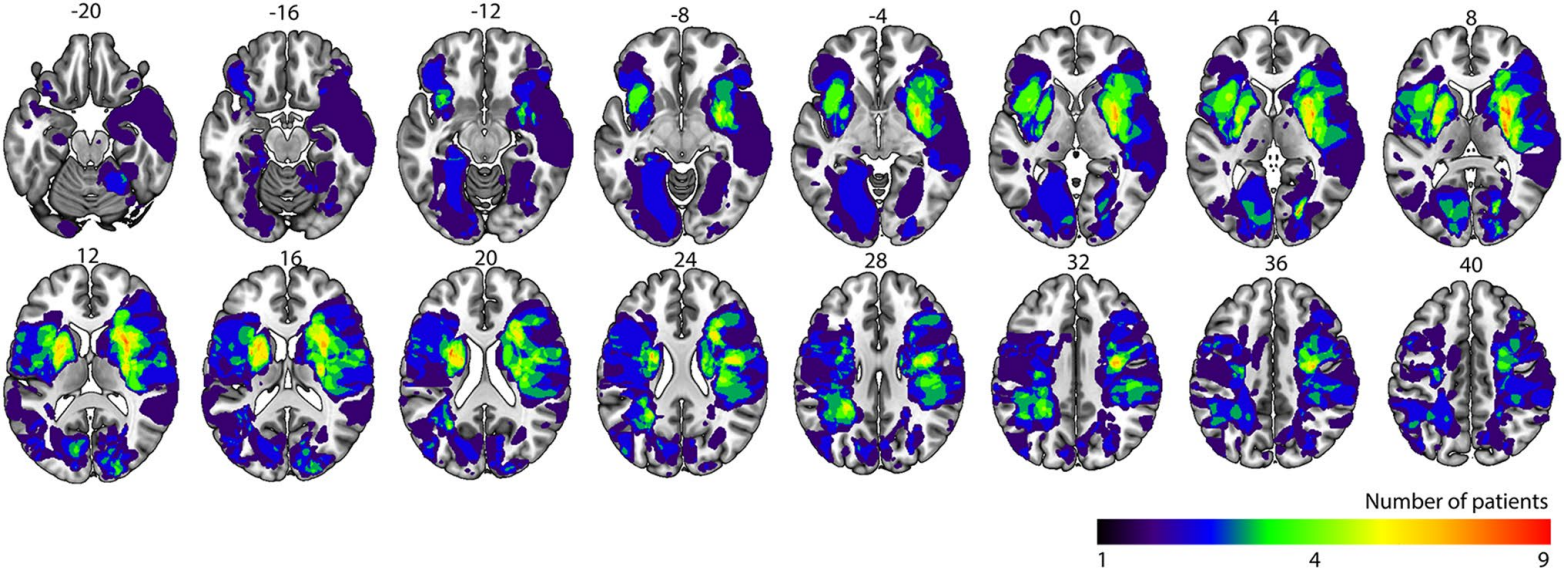
Lesion prevalence map as an overlay on the 1 mm MNI-152 template. Numbers above the slices correspond with z-coordinates in MNI space. Left hemisphere is depicted on the left. The color bar indicates the number of patients with a lesion for each voxel. Voxels that are lesioned in at least four patients, green colors and warmer, are included in the LSM analyses. Maximum overlap is 9

Median lesion volume was 5.77 cm^3^ (range 0.79–137.49 cm^3^). Figure 3 shows the lesion prevalence map, voxels lesioned in at least four patients have a green, yellow or red color. Lesions in the left hemisphere are as frequent as in the right hemisphere (Table 1) although median lesion size is larger in the right hemisphere (6.16 versus 3.97 cm^3^).

The association between lesion volume and behavioral outcome measures was assessed with correlations and the significance of these correlations was assessed using permutation tests, after accounting for effects of age, education, interval between stroke and assessment, and scanner. For discriminability, there was a significant association with lesion volume with the 2-back task, *r* (81) = −0.22, *p* = 0.02, a larger lesion volume was associated with lower discriminability. For the subsequent memory task, there was no significant correlation, *r *(79) = −0.12, *p* = 0.14. For the working memory task, there was no significant association between absolute response bias and total lesion volume, *r* (81) = 0.10, *p* = 0.17. The correlation between absolute response bias on the subsequent memory task and lesion volume was significant, *r* (79) = 0.32, *p* = 0.01. Larger lesion volume was associated with a more conservative response bias indicated by a significant positive correlation between the continuous measure of response bias and lesion volume, *r* (79) = 0.32, *p* < 0.01. Results remained the same after exclusion of one patient with a significantly larger lesion volume (> 3 SD).

### Multivariate lesion symptom mapping

**Fig. 4:**
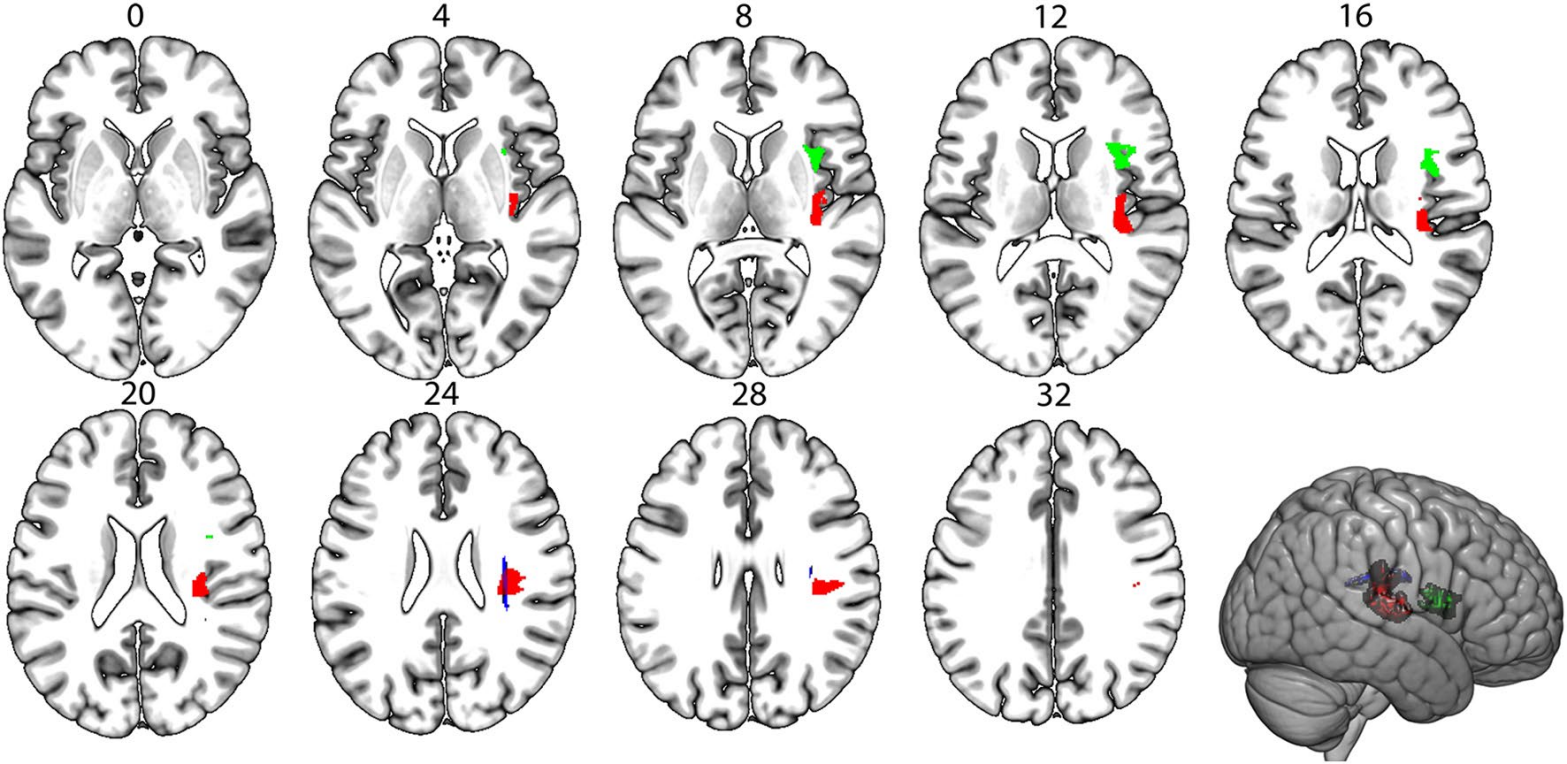
Results from the multivariate LSM analysis, controlled for age, education, interval between stroke and assessment, and scanner, but not for lesion volume. In red the cluster for discriminability on the 2-back task, in green the cluster associated with criterion setting on the subsequent memory task. Results from the atlas-based LSM analysis for 2-back discriminability, controlled for age, education, interval between stroke and assessment, and scanner, uncorrected for lesion volume in blue. Numbers refer to MNI coordinates, the left hemisphere is depicted on the left

Analyses identified for discriminability on the 2-back task, a cluster that based on the CAT atlas overlaps with the anterior and long segment of the arcuate fasciculus in the right hemisphere (voxelwise threshold *p* < 0.005, cluster size 2277, peak voxel: *x* = 37, *y* = −14, *z* = 9, clusterwise *p* = 0.02). This effect remained significant when discriminability on the subsequent memory task was added as covariate (voxelwise threshold *p* < 0.005, cluster size 2277, peak voxel: *x* = 37, *y* = −14, *z* = 9, clusterwise *p* = 0.02, Fig. 4a). When lesion volume was corrected for, the association was no longer significant (*p* = 0.08, with subsequent memory as covariate *p* = 0.06). For discriminability on the subsequent memory task, and criterion on the 2-back task, no significant clusters were identified. For criterion setting on the subsequent memory task a cluster in the right hemisphere was identified that overlapped with the frontal operculum on the GLASSER atlas (voxelwise threshold *p* < 0.005, cluster size 1017, peak voxel: *x* = 36, *y* = 4, *z* = 8, clusterwise *p* = 0.04). This association remained when adding criterion on the 2-back task (voxelwise threshold *p* < 0.005, cluster size 1198, peak voxel: *x* = 36, *y* = 4, *z* = 8, clusterwise *p* = 0.04). When lesion volume was corrected for the association was no longer significant (*p* = 0.17, with 2-back criterion as covariate *p* = 0.18) All analyses were controlled for age and education, interval between stroke and assessment, and scanner.

### Atlas-based lesion symptom mapping

Of the 360 Gy-matter ROIs included in the corrected GLASSER atlas, 111 were covered by at least 4 lesions. The white-matter CAT atlas consists of 32 ROIs out of which 28 had sufficient lesion coverage (see Supplemental Table S1 for details). For discriminability on the 2-back task, a significant correlation was found with lesion status after controlling for age, education, interval between stroke and assessment, and scanner. Lesion status in the long segment of the arcuate fasciculus in the right hemisphere was associated with 2-back discriminability (*z* = −3.27, threshold *z* < −3.16, Fig. 4), this effected is based on eight patients with a lesion in this tract. This effect remained no longer significant when discriminability on the subsequent memory task was added as covariate, nor when lesion volume was corrected for. For the other behavioral measures, no association with lesion status based on the atlas-based analyses. Age and education level did not correlate significantly with lesion status in any of the ROIs.

### Track-based lesion symptom mapping

**Fig. 5:**
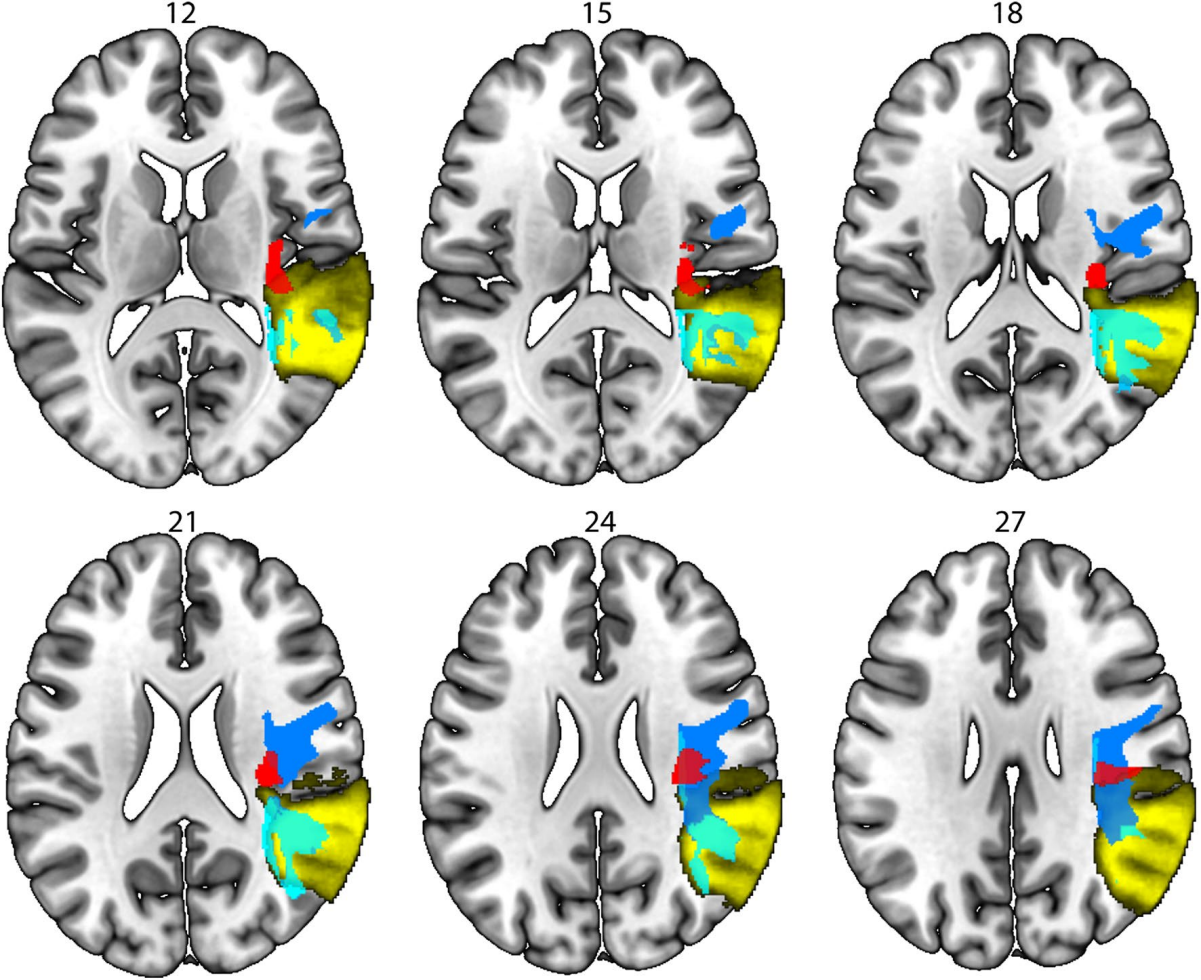
Results from the multivariate LSM for discriminability on the working memory task (in red) overlaid on the three segments from the arcuate fasciculus based on the binary CAT-atlas (in blue from dark to light: anterior segment, long segment, posterior segment) and the probabilistic atlas used by Tractotron (posterior segment in yellow)

To further investigate the role of the right arcuate fasciculus in working-memory discriminability, we conducted track-based analysis for the anterior, long, and posterior segments in the right hemisphere. The probability of disconnection was above chance level in 22 patients for the anterior segment of the arcuate fasciculus, in 21 patients for the long segment, and in ten patients for the posterior segment. The results of a Mann–Whitney *U* test revealed that patients with a disconnection in the posterior segment of the arcuate fasciculus had lower discriminability on the 2-back task (Mdn = −1.14) than patients with an intact posterior segment (Mdn = 0.13, *U* = 207, *p* = 0.03). The proportion of disconnection of the posterior segment was negatively correlated with discriminability on the 2-back task, *r* (81) = −0.27, *p* = 0.01. Significance of the correlation was assessed by permutation testing. The effect remained when including discriminability on the subsequent memory task as covariate, *r* (79) = −0.27, *p* < 0.01. To check whether the posterior segment of the arcuate fasciculus is uniquely negatively associated with discriminability in working memory, we conducted the same test for discriminability on the subsequent memory test. Patients with the posterior segment disconnected had a *higher* discriminability than the intact group (Mdn = 0.67 compared to Mdn = −0.12, *U* = 207, *p* = 0.04). The correlation with severity was not significant (*r* (79) = 0.19, *p* = 0.09). As the a-priori hypothesis is that lesions do not result in better performance, this effect can be interpreted as having a lesion in the posterior segment of the arcuate fasciculus makes it more likely to not have a lesion in a region that is crucial for discriminability on the subsequent memory task. Both the behavioral and tract data were controlled for age, education, interval between stroke and assessment, and scanner. For the anterior and long segment of the arcuate fasciculus, there were no significant associations. The different results concerning the different segments of the arcuate are likely due to the difference between a binary atlas (CAT) and a probabilistic atlas (Tractotron). The cluster identified with the multivariate analyses overlaps with the anterior and long segment of the arcuate based on the CAT atlas, but the probabilistic atlas used by Tractotron shows that this cluster also overlaps with the posterior segment (Fig. 5).

## Discussion

The aim of this study was to contrast the theory of separate memory stores with the theory of working memory as activated long-term memory, by investigating the behavioral and neuroanatomical correlates of working and episodic memory in a stroke population. To this end, we used a task design in which working memory and episodic memory are assessed based on the same encoding phase. We used behavioral and neuroimaging data to investigate (1) the relation between visual working memory and episodic memory performance in stroke patients and older adults and (2) anatomical correlates of visual memory function using multivariate voxel-based, atlas-based, and track-based approaches. We found that discriminability in working memory and episodic memory were independent at the behavioral level. In contrast, response bias was correlated between working memory and episodic memory in stroke patients. LSM analyses suggested there might be independent regions that are associated with working memory and episodic memory performance.

The key issue in the ongoing debate on the multicomponent model of memory versus the view of working memory as activated long-term memory, is the need of a separate and independent short-term memory store (Baddeley et al. 2019; Cowan 2019; Norris 2017, 2019; Oberauer 2009; Shallice and Papagno 2019). According to the multicomponent model, a separate store and mechanism is needed to construct new representations and actively maintain relational information (Norris 2017, 2019). The theory of activated long-term memory states that this can be achieved by rapid new learning, in which new associations can be formed as new long-term memory trace. While the multicomponent model of memory explains long-term memory deficits as the failure to encode a representation into long-term memory, the theory of activated long-term memory interprets this as a failure of consolidation of rapidly formed new long-term memory traces (Cowan 2019). If rapidly formed representations underlie associative memory, interference or a deficit in consolidation explains low performance on the subsequent memory task but does not explain low performance solely on the working memory task. Our results suggest that there might be separate representations in working memory and episodic memory as discriminability is not correlated between the tasks and some patients show selective impairment. Response bias on the other hand might rely on common neural substrates in working memory and episodic memory as this does correlate between the tasks in stroke patients.

Results from the LSM analyses show independent regions that are stronger associated with working memory and episodic memory performance. Lesions in the arcuate fasciculus in the right hemisphere were more strongly associated with discriminability in working memory than in subsequent memory, while lesions in the frontal operculum in the right hemisphere were more strongly associated with criterion setting in subsequent memory than in working memory. As we included the scores for discriminability and criterion on the other task as covariate, we can state that there is a stronger association for one task than for the other with lesion status in these regions. The arcuate fasciculus connects the perisylvian cortex of the frontal, parietal, and temporal lobes. In the left hemisphere, the three segments of the arcuate form the perisylvian language network, which is extensively studied (e.g.,Bonakdarpour et al. 2019; Catani et al. 2005). The left anterior segment has been associated with the phonological loop, specifically with order errors (Papagno et al. 2017). The right arcuate fasciculus has been studied less extensively, but available studies associated lesions in this region with spatial neglect (Catani and de Schotten 2008; Machner et al. 2018), visuospatial processing (Rolland et al. 2018), and visual working memory (Chechlacz et al. 2014; Matias-Guiu et al. 2018). We found discriminability on the working memory task, compared to the episodic memory task, to be stronger related to lesions in the anterior and long segment of arcuate fasciculus based on multivariate and atlas-based analyses. Track-based analyses demonstrated an association with the posterior segment of the arcuate fasciculus only for discriminability on the working memory task but not the subsequent memory task. As the working memory task is based on temporal order, our findings converge with previous results for verbal order information in the left anterior segment of the arcuate fasciculus. The posterior segment of the arcuate fasciculus connects Wernicke’s areas to the inferior parietal lobe. Previous studies have identified the right inferior parietal lobe to be involved in reorienting attentional focus to memory representations of previously attended stimuli (Kizilirmak et al., 2015). Based on our results and previous findings we suggest that the right arcuate fasciculus might be associated with the visuospatial sketchpad. The different results concerning the different segments of the arcuate fasciculus are likely due to the difference between a binary atlas (CAT) and a probabilistic atlas (Tractotron). The cluster identified with the multivariate analyses overlaps with the anterior and long segment of the arcuate fasciculus based on the CAT atlas, but the probabilistic atlas used by Tractotron shows that this cluster also overlaps with the posterior segment. All analyses indicate that the right arcuate fasciculus is more involved in working memory than subsequent memory. DTI analyses in a future study should give more insight in the role of different segments of the right arcuate fasciculus in working memory. As track-based analyses provide better evidence for behavioral correlations for white matter lesions, this might be an indication that specifically the posterior segment of the right arcuate fasciculus is essential for visual working memory.

Criterion setting was stronger associated with the frontal operculum for subsequent memory compared to working memory. It is interesting to note that for criterion setting we only found an association with lesion status in the frontal operculum for the subsequent memory task while criterion was correlated between the two tasks at the behavioral level. Even though the correlation was statistically significant, the correlation was weak. The correlation might be explained by a third factor influencing response bias on both tasks even if they have different neural substrates. A possible factor related to response bias is age (for a meta-analysis, see Fraundorf et al., 2019). The frontal operculum has been described as essential in exerting control over cognitive processes (Takayasu et al., 2011). It was shown to be related to selective attention and to regulate activity in occipitotemporal areas involved in the processing of different classes (faces, houses, bodies) of visual stimuli (Takayasu et al., 2011). A second study found evidence for activation of the frontal operculum during interference tasks that required response inhibition (Wager et al., 2005). A third study showed that resisting bias based on irrelevant previous information was associated with activation of the frontal operculum (Scholl et al., 2015). These findings converge with our results which suggest that damage to the frontal operculum might result in a stronger response bias.

The results from our LSM analyses should be interpreted with caution because the associations between memory performance and lesion location were no longer significant after correction for lesion volume. Larger lesion volume was associated with lower discriminability on the working memory task and stronger response bias in the subsequent memory task. The finding that lesion volume is associated with performance does not nullify the result that specific regions in the brain are stronger related with the one memory task compared to the other.

Our results partly converge with a previous study in stroke patients on discriminability and criterion setting in verbal recognition memory. Like in our study, Biesbroek et al. (2015) reported that the right inferior frontal gyrus/frontal operculum is crucial for criterion setting. This study indicated that the left medial temporal lobe, left temporo‐occipital structures, both thalami, and the right hippocampus are associated with discriminability (Biesbroek et al. 2015). Two main differences should be pointed out, the verbal versus visual nature of the task and the distribution of lesions. Lesion symptom-mapping studies rely heavily on the total lesion prevalence distribution resulting in differences between studies. Previous studies have shown different neural correlates for verbal and visual memory (e.g., Donolato et al. 2017).

The advantage of studying stroke patients is that due to the sudden nature of the brain damage, it is acceptable to infer causal relations (Karnath et al. 2019; Rorden and Karnath 2004). A critical comment is that people with stroke might have a higher vascular burden that is related to memory function (Van Leijsen et al. 2019). There might be a selection bias in the sample with patients with mild symptoms and small lesions being more likely to participate in research. This has a consequence for the distribution of lesions across the brain, though this is party inherent to the population studied. Brain lesions due to stroke are determined by the vascular tree resulting in vulnerable lesion sites and intercorrelation between voxels. Even though there might be locations in the brain crucial to a specific task that are rarely affected by stroke, these would not be considered as main associates for post-stroke memory deficits. A limitation remains that we can only draw conclusions on the voxels/ROIs with sufficient lesion coverage and that some areas typically associated with memory, like the hippocampus, were not included in the analyses.

The aim of the study was to investigate how stroke patients can give insight into shared and distinct processes in working memory and episodic memory. Due to the limited lesion coverage that is, however, typical for a stroke sample (Zhao et al. 2018), we cannot make any claims on hippocampal/medial temporal lobe structures that may or may not be involved in both visual working memory and episodic memory. However, the current study does give an indication that other brain regions are also associated with working memory performance (the right arcuate fasciculus) and with criterion setting in episodic memory (the right frontal operculum). Furthermore, the behavioral data provide moderate evidence that discriminability in visual working memory and episodic memory are unrelated, supported by a lack of correlation and by selective impairments. They also give strong evidence that criterion setting in working memory and episodic memory are correlated.

With the task design we used, we aimed to assess working memory and episodic memory in one task design, using the same stimuli, the same encoding phase and comparable binding demands. The difficulty is assessing two different processes in a comparable task with limited confounding factors differentiating between which processed is tapped into. It is important to stress that both tasks involve context binding. The 2-back task is a temporal order binding task, so not based solely on object recognition as all objects appeared twice in the same block. The subsequent memory task assessed spatial binding. A few limitations concerning the task design should be mentioned. First, although several studies indicate that contextual binding for time and space relies on the hippocampus (e.g.,Eichenbaum 2017; Yonelinas et al. 2019), they might not have fully overlapping neural correlates. A recent study showed that different subregions within the hippocampus were differently associated with object-location, object-time and object-object associations in development from childhood into adolescence based on structural MRI in 171 subjects (Lee et al. 2020). Furthermore, an fMRI study of 16 healthy subjects showed activations in specific areas for spatial order (parahippocampus) and temporal order processing (Brodmann area 10 within the prefrontal cortex), in addition to general hippocampal involvement for source retrieval (Ekstrom et al. 2011). Given these results, we cannot fully rule out the possibility of stroke selectively affecting different types of binding. Second, hyper-binding might differently affect the working memory and subsequent memory task. In the ageing literature, hyper-binding refers to the inability of older adults to inhibit irrelevant information resulting in lower performance on a working memory task but enhanced performance when the previously irrelevant information is subsequently tested (e.g. Campbell et al. 2010). However, in our design, we do not expect this to have a large influence. Even though location was not relevant during the 2-back task, the information was not conflicting and could even be used as a cue as a target could only be in the same location as two trials previously. Secondly, hyper-binding only occurs under fully implicit instructions (Campbell and Hasher 2018). In our task, participants are made explicitly aware of the link between the tasks. Campbell and Hasher (2018) showed that the effect of hyper-binding in older adults disappears when made aware of the connection between the tasks. Finally, our previous study in which we studied the effect of age on memory with this task design, did not show an advantage for older adults on the subsequent memory task (for a more extensive discussion on the task design see Lugtmeijer et al. 2019). A third difference is in task encoding, the subsequent memory task is unexpected. While encoding for working memory is typically shallow and based on rehearsal, encoding for a planned long-term retention task is more elaborative, which is beneficial for episodic memory but less essential for working memory (Cowan 2019; Craik and Watkins 1973). While this might result in associations with different neural substrates than typically found in explicit episodic memory tasks, these instructions ensure that the encoding strategy is not different between the tasks. Therefore, this design is more sensitive to detecting possible overlapping substrates for working memory and episodic memory. A second possible difference in encoding might be verbalization. The working memory task can be supported by verbal labelling of the objects (e.g., apple, car, apple). The participants were instructed that the second appearance of an object was always on the same location as the first so location could be used as a cue (e.g., apple right lower corner) but as location was irrelevant to the working memory task, it is unknown whether participants included that in their verbal label. Furthermore, our LSM analyses do not indicate a dominant role for verbalization. We identified right hemispheric counter parts of typical language areas to be associated with working memory performance.

For clinical cognitive assessment, it is relevant to take into account that stroke patients might have an altered response bias, especially because our results show that stroke can affect response bias towards a more liberal and a more conservative bias.

In conclusion, stroke can result in both working memory and episodic memory deficits. This study indicates that discriminability in working memory and episodic memory are two distinct processes, while criterion setting might be a shared process. LSM analyses suggested that independent regions are stronger associated with visual working memory (right arcuate fasciculus) and criterion setting in episodic memory (frontal operculum).

## Supplementary Information

Below is the link to the electronic supplementary material.

Supplementary file1 (PDF 192 KB)

## Data Availability

Toolboxes used for lesion analysis (NiiStat and SVR-LSM GUI) are freely available at GitHub. Adjusted code is available upon request.

## Availability of data and material

Anonymized data are available upon request a year after the completion of the project (01-07-2021).
