# Supplementary material for: Are visual working memory and episodic memory distinct processes? Insight from stroke patients by lesion-symptom mapping
Source: Brain Struct Funct. 2021 Apr 29;226(6):1713–26. doi: 10.1007/s00429-021-02281-0 (PMC8203519; doi:10.1007/s00429-021-02281-0)
Supplement: Supplementary file 1 — Supplementary file1 (PDF 192 KB) [file 429_2021_2281_MOESM1_ESM.pdf]

Are visual working memory and episodic memory distinct processes? Insight from stroke patients by lesion-symptom mapping.

Brain Structure and Function

Selma Lugtmeijer (1,2), Linda Geerligs (1), Frank Erik de Leeuw (3), Edward H. F. de Haan (2), Roy P. C. Kessels (1,4), on behalf of The Visual Brain Group

(1) Donders Institute for Brain, Cognition and Behaviour, Radboud University, Nijmegen, the Netherlands; (2) University of Amsterdam, the Netherlands; (3) Radboud University Medical Center, Department of Neurology, Nijmegen, the Netherlands; (4) Radboud University Medical Center, Department of Medical Psychology, Nijmegen, the Netherlands.

Corresponding author: R. Kessels - r.kessels@donders.ru.nl

Supplemental Table S1

List of number of subjects with a lesion per ROI. Only ROIs in which at least four subjects had a lesion were included in the atlas-based analyses.

GLASSER atlas			Left	Right
1	V1	Primary Visual Cortex	6	9
2	MST	Medial Superior Temporal Area	3	0
3	V6	Sixth Visual Area	3	1
4	V2	Second Visual Area	6	9
5	V3	Third Visual Area	7	7
6	V4	Fourth Visual Area	6	10
7	V8	Eighth Visual Area	4	4
8	4	Primary Motor Cortex	6	12
9	3b	Primary Sensory Cortex	2	6
10	FEF	Frontal Eye Fields	1	4
11	PEF	Premotor Eye Fields	4	6
12	55b	Area 55b	3	4
13	V3A	Area V3A	1	4
14	RSC	RetroSplenial Complex	2	0
15	POS2	Parieto-Occipital Sulcus Area 2	4	4
16	V7	Seventh Visual	1	3
17	IPS1	Intraparietal Sulcus Area 1	2	3
18	FFC	Fusiform Face Complex	3	3
19	V3B	Area V3B	3	4
20	LO1	Area Lateral Occipital 1	1	1
21	LO2	Area Lateral Occipital 2	1	0

22	PIT	Posterior Inferotemporal Complex	2	2
23	MT	Middle Temporal Area	4	0
24	A1	Primary Auditory Cortex	4	4
25	PSL	Perisylvian Language Area	2	5
26	SFL	Superior Frontal Language Area	0	1
27	PCV	Precuneus Visual Area	2	2
28	STV	Superior Temporal Visual Area	2	2
29	7Pm	Medial Area 7P	3	3
30	7m	Area 7m	5	3
31	POS1	Parieto-Occipital Sulcus Area 1	4	2
32	23d	Area 23d	1	0
33	v23ab	Area ventral 23 a+b	1	0
34	d23ab	Area dorsal 23 a+b	0	0
35	31pv	Area 31p ventral	2	0
36	5m	Area 5m	2	0
37	5mv	Area 5m ventral	4	1
38	23c	Area 23c	4	0
39	5L	Area 5L	0	1
40	24dd	Dorsal Area 24d	3	0
41	24dv	Ventral Area 24d	1	0
42	7AL	Lateral Area 7A	2	2
43	SCEF	Supplementary and Cingulate Eye Field	1	1
44	6ma	Area 6m anterior	0	1
45	7Am	Medial Area 7A	3	2
46	7PL	Lateral Area 7P	0	1
47	7PC	Area 7PC	3	2
48	LIPv	Area Letral Intraparietal ventral	5	5
49	VIP	Ventral Intraparietal Complex	3	1
50	MIP	Medial Intraparietal Area	2	3
51	1	Area 1	3	4
52	2	Area 2	5	3
53	3a	Area 3a	3	9
54	6d	Dorsal Area 6	2	3
55	6mp	Area 6mp	2	0
56	6v	Ventral Area 6	3	6
57	p24pr	Area Posterior 24 prime	1	0
58	33pr	Area 33 prime	1	0
59	a24pr	Anterior 24 prime	1	0
60	p32pr	Area p32 prime	1	0
61	a24	Area 24a	0	0
62	d32	Area dorsal 32	0	0
63	8BM	Area 8BM	0	0
64	p32	Area p32	0	0
65	10r	Area 10r	0	0
66	47m	Area 47m	2	1
67	8Av	Area 8Av	3	7
68	8Ad	Area 8Ad	1	0
69	9m	Area 9 Middle	0	0
70	8BL	Area 8B Lateral	0	0
71	9p	Area 9 Posterior	0	0
72	10d	Area 10d	0	0
73	8C	Area 8C	5	6
74	44	Area 44	4	5
75	45	Area 45	4	5
76	47l	Area 47l	4	5

77	a47r	Area anterior 47r	2	1
78	6r	Rostral Area 6	6	8
79	IFJa	Area IFJa	4	6
80	IFJp	Area IFJp	6	6
81	IFSp	Area IFSp	2	5
82	IFSa	Area IFSa	2	3
83	p9-46v	Area posterior 9-46v	2	4
84	46	Area 46	1	4
85	a9-46v	Area anterior 9-46v	1	2
86	9-46d	Area 9-46d	1	1
87	9a	Area 9 anterior	0	0
88	10v	Area 10v	0	0
89	a10p	Area anterior 10p	0	0
90	10pp	Polar 10p	0	0
91	11l	Area 11l	2	1
92	13l	Area 13l	1	2
93	OFC	Orbital Frontal Complex	0	0
94	47s	Area 47s	4	3
95	LIPd	Area Lateral Intraparietal dorsal	3	3
96	6a	Area 6 anterior	2	5
97	i6-8	Inferior 6-8 Transitional Area	1	5
98	s6-8	Superior 6-8 Transitional Area	0	1
99	43	Area 43	6	6
100	OP4	Area OP4/PV	4	4
101	OP1	Area OP1/SII	5	6
102	OP2-3	Area OP2-3/V3	6	7
103	52	Area 52	5	5
104	RI	Retroinsular Cortex	3	5
105	PFcm	Area PFcm	8	6
106	PoI2	Posterior Insular Area 2	8	12
107	TA2	Area TA2	0	6
108	FOP4	Frontal OPercular Area 4	6	7
109	MI	Middle Insular Area	5	8
110	Pir	Pirform Cortex	7	6
111	AVI	Anterior Ventral Insular Area	4	7
112	AAIC	Anterior Agranular Insular Complex	5	6
113	FOP1	Frontal OPercular Area 1	5	6
114	FOP3	Frontal OPercular Area 3	5	8
115	FOP2	Frontal OPercular Area 2	4	8
116	PFt	Area PFt	3	3
117	AIP	Anterior Intraparietal Area	4	5
118	EC	Enthorhinal Cortex	1	1
119	PreS	Presubiculum	3	1
120	H	Hippocampus	4	1
121	ProS	Prostriate Area	2	1
122	PeEc	Perirhinal Ectorhinal Cortex	0	1
123	STGa	Area STGa	2	1
124	PBelt	ParaBelt Complex	3	6
125	A5	Auditory 5 Complex	0	2
126	PHA1	Parahippocampal Area 1	3	1
127	PHA3	Parahippocampal Area 3	2	2
128	STSda	Area STSd anterior	1	1
129	STSdp	Area STSd posterior	2	2
130	STSvp	Area STSv posterior	1	2
131	TGd	Area TG dorsal	2	1

132	TE1a	Area TE1 anterior	1	1
133	TE1p	Area TE1 posterior	0	2
134	TE2a	Area TE1 anterior	1	1
135	TF	Area TF	0	2
136	TE2p	Area TE2 posterior	1	1
137	PHT	Area PHT	3	2
138	PH	Area PH	2	1
139	TPOJ1	Area Temporoparietooccipital Junction 1	2	3
140	TPOJ2	Area Temporoparietooccipital Junction 2	3	1
141	TPOJ3	Area Temporoparietooccipital Junction 3	4	1
142	DVT	Dorsal Transitional Visual Area	5	5
143	PGp	Area PGp	3	1
144	IP2	Area Intraparietal 2	6	4
145	IP1	Area Intraparietal 1	3	3
146	IP0	Area Intraparietal 0	3	5
147	PFop	Area PF opercular	6	4
148	PF	Area PF Complex	6	3
149	PFm	Area PFm Complex	5	3
150	PGi	Area PGi	6	2
151	PGs	Area PGs	4	1
152	V6A	AreaV6A	0	1
153	VMV1	Ventromedial Visual Area 1	2	1
154	VMV3	Ventromedial Visual Area 3	2	1
155	PHA2	Parahippocampal Area 2	2	1
156	V4t	Area V4t	3	0
157	FST	Area FST	3	1
158	V3CD	Area V3CD	3	3
159	LO3	Area Lateral Occipital 3	4	0
160	VMV2	Ventromedial Visual Area 2	2	1
161	31pd	Area 31pd	3	1
162	31a	Area 31a	0	0
163	VVC	Ventral Visual Complex	2	3
164	25	Area 25	0	0
165	s32	Area s32	0	0
166	pOFC	Posterior OFC Complex	1	1
167	PoI1	Area Posterior Insular 1	7	9
168	Ig	Insular Granular Complex	6	9
169	FOP5	Area Frontal Opercular 5	4	5
170	p10p	Area posterior 10p	0	0
171	p47r	Area posterior 47r	1	2
172	TGv	Area TG ventral	1	1
173	MBelt	Medial Belt Complex	1	7
174	LBelt	Lateral Belt Complex	4	4
175	A4	Auditory 4 Complex	3	4
176	STSva	Area STSv anterior	2	1
177	TE1m	Area TE1 Middle	0	1
178	PI	Para-Insular Area	2	5
179	a32pr	Area anterior 32 prime	0	0
180	p24	Area posterior 24	0	0

CAT atlas		Left	Right
1	Anterior Commissure	5	7
2	Anterior Segment Arcuate Fasciculus	13	14
3	Long Segment Arcuate Fasciculus	14	8
4	Posterior Segment Arcuate Fasciculus	12	6

5	Cingulum	14	16
6	Corpus Callosum	28	34
7	Corticoponto Cerebellum	21	19
8	Corticospinal Tract	32	27
9	Fornix	7	6
10	Inferior Cerebellar Pedunculus	1	3
11	Inferior Longitudinal Fasciculus	16	11
12	Inferior Occipitofrontal Fasciculus	15	21
13	Internal Capsule	30	28
14	Optic Radiations	18	13
15	Superior Cerebelar Pedunculus	0	2
16	Uncinate	9	10

References

Catani M, De Schotten MT. 2008. A diffusion tensor imaging tractography atlas for virtual in vivo dissections. *Cortex.* 44:1105-1132. DOI: 10.1016/j.cortex.2008.05.004

Glasser MF, Coalson TS, Robinson EC, Hacker CD, Harwell J, Yacoub E et al. 2016. A multi-modal parcellation of human cerebral cortex. *Nature.* 536:171. DOI: 10.1038/nature18933
Supplementary Neuroanatomical Results
